# Gβγ mediates activation of Rho guanine nucleotide exchange factor ARHGEF17 that promotes metastatic lung cancer progression

**DOI:** 10.1016/j.jbc.2021.101440

**Published:** 2021-11-20

**Authors:** Irving García-Jiménez, Rodolfo Daniel Cervantes-Villagrana, Jorge Eduardo del-Río-Robles, Alejandro Castillo-Kauil, Yarely Mabell Beltrán-Navarro, Jonathan García-Román, Guadalupe Reyes-Cruz, José Vázquez-Prado

**Affiliations:** 1 Department of Cell Biology, CINVESTAV-IPN, Mexico City, Mexico; 2 Department of Pharmacology, CINVESTAV-IPN, Mexico City, Mexico

**Keywords:** ARHGEF17, Gbetagamma, lysophosphatidic acid, heterotrimeric G protein, RhoA, Rho guanine nucleotide exchange factor, actin cytoskeleton, signal transduction, cell migration, DMEM, Dulbecco's modified Eagle's medium, EGFP, enhanced GFP, ENPP2, ectonucleotide pyrophosphatase/phosphodiesterase 2, ERK, extracellular-regulated kinase, FBS, fetal bovine serum, GPCR, G protein–coupled receptor, GST, glutathione-*S*-transferase, HEK 293T, human embryonic kidney 293T cell line, LPA, lysophosphatidic acid, LPAR, lysophosphatidic acid receptor, NSCLC, non–small cell lung carcinoma, PAE, porcine aortic endothelial, PTX, pertussis toxin, RhoGEF, Rho guanine nucleotide exchange factor

## Abstract

Metastatic lung cancer is a major cause of death worldwide. Dissemination of cancer cells can be facilitated by various agonists within the tumor microenvironment, including by lysophosphatidic acid (LPA). We postulate that Rho guanine nucleotide exchange factors (RhoGEFs), which integrate signaling cues driving cell migration, are critical effectors in metastatic cancer. Specifically, we addressed the hypothetical role of ARHGEF17, a RhoGEF, as a potential effector of Gβγ in metastatic lung cancer cells responding to LPA. Here, we show that ARHGEF17, originally identified as a tumor endothelial marker, is involved in tumor growth and metastatic dissemination of lung cancer cells in an immunocompetent murine model. Gene expression–based analysis of lung cancer datasets showed that increased levels of ARHGEF17 correlated with reduced survival of patients with advanced-stage tumors. Cellular assays also revealed that this RhoGEF participates in the invasive and migratory responses elicited by Gi protein–coupled LPA receptors *via* the Gβγ subunit complex. We demonstrate that this signaling heterodimer promoted ARHGEF17 recruitment to the cell periphery and actin fibers. Moreover, Gβγ allosterically activates ARHGEF17 by the removal of inhibitory intramolecular restrictions. Taken together, our results indicate that ARHGEF17 may be a valid potential target in the treatment of metastatic lung cancer.

Lipid-derived agonists and chemokines within the tumor microenvironment attract stromal cells and promote dissemination of cancer cells (1, 2, 3). They stabilize the active conformation of chemotactic G protein–coupled receptors (GPCRs) to engage an intracellular repertoire of G protein–dependent and independent mechanisms (4, 5). During cell invasion and migration, GPCRs drive actin cytoskeleton reorganization through Rho GTPases, such as RhoA, Rac1, and Cdc42. These GTPases are activated by multidomain signaling proteins called Rho guanine nucleotide exchange factors (RhoGEFs) (6, 7, 8). Specifically, these multidomain effectors are key signaling proteins, and potential therapeutic targets, that set with precision where and which Rho GTPases are loaded with GTP, acquiring an active conformation that control the assembly of different kinds of actin filaments and actomyosin contractile complexes (9, 10, 11, 12). Although reorganization of the actin cytoskeleton by RhoGTPases is a ubiquitous mechanism for cell migration, the signaling proteins upstream of these processes are quite diverse. The involved repertoire of signaling molecules includes distinct families of heterotrimeric G proteins, particularly Gα12/13, Gαq/11, and Gβγ subunits, some of which have been linked to cancer metastasis and drug resistance (13, 14, 15, 16, 17, 18, 19); as well as multiple RhoGEFs, that according to their position within signaling cascades, are key to drive cell migration under aberrant conditions such as metastatic cancer (9, 20). Examples of RhoGEFs mechanistically linked to dissemination of various cancer cell types include DOCK1, DOCK3, P-Rex1, PDZ-RhoGEF, ARHGEF5, ARHGEF7, GEF-H1, NET1, Vav2/3, Tiam1, and Trio (21, 22, 23, 24, 25, 26, 27, 28, 29, 30, 31, 32, 33, 34). Therefore, RhoGEFs are intermediaries of migratory pathways activated by cancer and stromal cells in response to chemokines and lipid-derived agonists, such as lysophosphatidic acid (LPA) (35, 36, 37, 38, 39, 40).

Within the tumor microenvironment, LPA is produced and subjected to changes on its spatiotemporal availability, creating self-generated gradients that sustain directional migration of various cancer cell types (41, 42, 43). Even though LPA is the cognate agonist of six different GPCRs; in oncogenic settings, LPA receptor 1 (LPAR1) has been highlighted as the main promoters of metastatic dissemination of lung, ovarian, pancreatic, melanoma, and breast cancer cells, among others (41, 42, 44, 45, 46, 47). Cell invasion driven by LPA occurs *via* Gi-dependent dynamic cytoskeleton remodeling that guides the assembly of actin fibers into invadopodia, delimiting areas of extracellular matrix degradation (48, 49).

We previously demonstrated that metastatic LAP0297 lung cancer cells coinoculated with bone marrow–derived cells exhibited an increased tumorigenic potential in immunocompetent mice (50). In response to LPA, these cells migrate *via* Gi-coupled receptors, pointing to G protein–regulated RhoGEFs as putative Gβγ effectors that integrate migratory cues (50). In this regard, ARHGEF17 (also known as TEM4/p164-RhoGEF), a RhoGEF potentially linked to tumor-induced angiogenesis (51, 52), has been found overexpressed in murine Lewis lung carcinoma tumors (53). This RhoGEF maintains intercellular adhesions in endothelial cell monolayers; whereas in migrating cells, it sustains persistent direction (54, 55, 56). Interestingly, the expression of ARHGEF17 is regulated by the Hippo pathway, and it is part of a transcriptional signature that, together with 21 other genes, shows prognostic value among various cancer types (57, 58). ARHGEF17 contains an actin-binding site through which it might regulate its activity during cell migration (59). Besides its RhoGEF activity, ARHGEF17 acts as a spindle assembly checkpoint timer, regulating mitotic fidelity *via* positioning of the kinetochore-associated kinase Mps1 (60). Since cell migration and proper control of the cell cycle are altered in cancer cells, the finding that ARHGEF17 is mechanistically linked to these essential processes is consistent with a putative dysregulation of this RhoGEF in carcinogenesis and metastasis. Although ARHGEF17 was originally identified as a tumor endothelial transcript (51), its potential role in cancer progression remains unknown. Since increased expression of ARHGEF17 correlated with bad prognosis in lung cancer patients with high-grade tumors, here we analyzed its role in tumor growth and metastasis. We used immunocompetent mice as a preclinical model to address the involvement of ARHGEF17 in tumorigenesis by syngeneic LAP0297 lung cancer cells and established the mechanism by which Gi-coupled LPARs activate this RhoGEF.

## Results

### Tumorigenic role of ARHGEF17, a RhoGEF that correlates with reduced survival of non–small cell lung carcinoma patients with high-grade tumors

**Figure 1:**
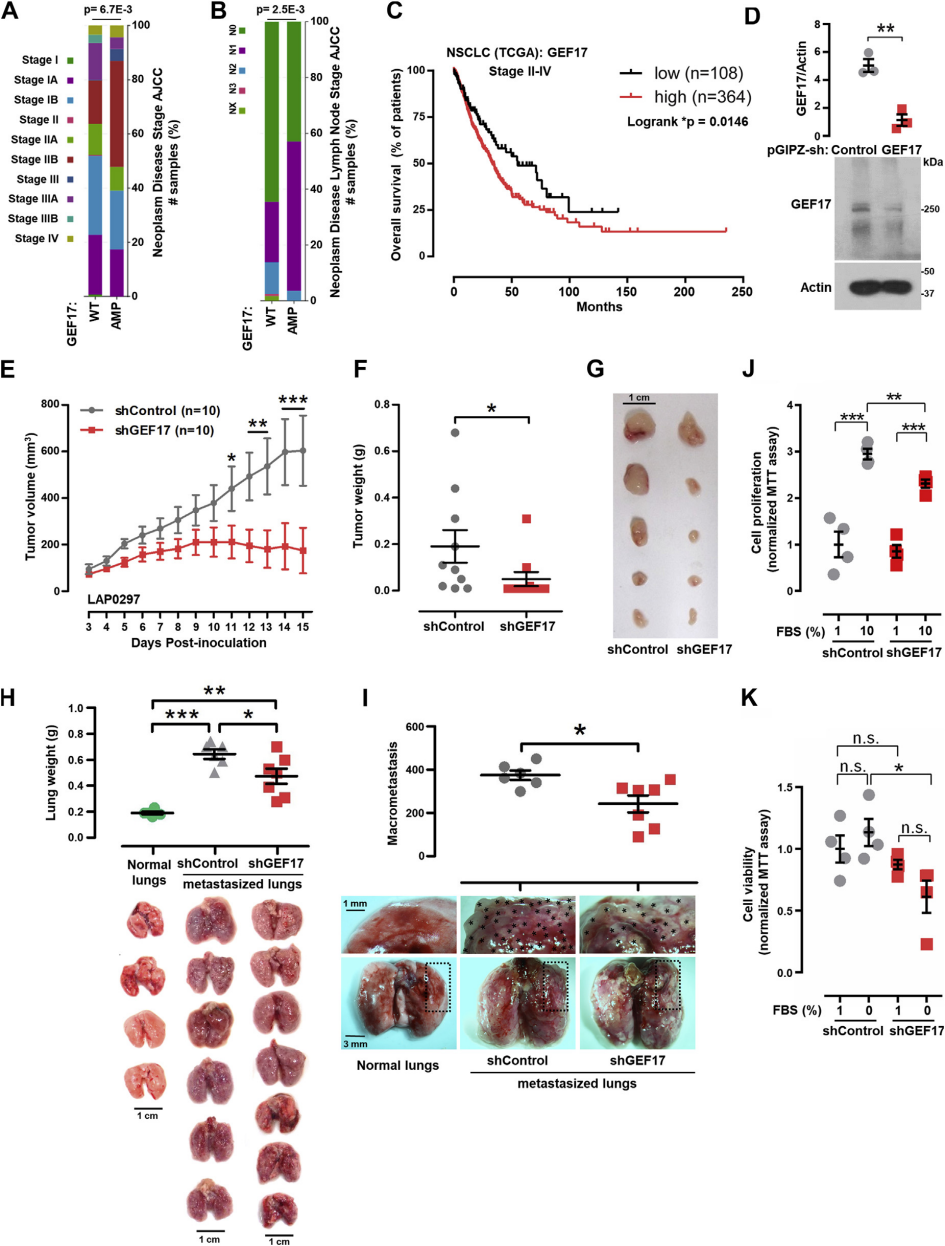
**Tumorigenic role of ARHGEF17.***A* and *B*, NSCLC TCGA patients with ARHGEF17 amplification (GEF17; AMP) compared with WT according to their tumor stage (*A*, stages I–IV) and lymph node disease (*B*, N0-3,NX) were analyzed by Chi-squared test at the cBioportal platform. *C*, maximally separated Kaplan–Meier plots of NSCLC (TCGA datasets) compared the survival of patients with high and low GEF17 expression (stages II–IV). *D*, GEF17 knockdown in LAP0297 cells. A representative Western blot is shown. Graph represents mean ± SEM, n = 3. ∗∗*p* < 0.01; *t* test. *E*, LAP0297 tumor growth in immunocompetent FVB mice inoculated with ARHGEF17-knockdown (sh-GEF17) or control cells (sh-Control). Graph represents the mean ± SEM; ∗*p* < 0.05, ∗∗*p* < 0.01, and ∗∗∗*p* < 0.001; ANOVA followed by Tukey's test, n = 10. *F*, tumor weight was measured at day 15. Graph represents the mean ± SEM; ∗*p* < 0.05; *t* test. n = 10. *G*, representative tumors are shown. The scale represents 1 cm. *H*, lung weight. Graph shows the mean ± SEM weight of normal lungs from healthy mice and metastasized lungs from mice inoculated in the tail vein with sh-Control or sh1-GEF17 LAP0297 cells. ∗*p* < 0.05, ∗∗*p* < 0.01, and ∗∗∗*p* < 0.001; Mann–Whitney *U* test. n = 6 control groups, n = 7 sh-GEF17. The scale represents 1 cm. *I*, macrometastasis in the lungs of FVB mice inoculated in the tail vein with sh-Control or sh1-GEF17 LAP0297 cells. Graph represents the number of superficial metastases, mean ± SEM; ∗*p* < 0.05; *t* test. n = 6 sh-Control, n = 7 sh-GEF17. Normal lungs from healthy mice are shown at the *left*. The scale represents 3 mm, zoom: 1 mm. *J* and *K*, *in vitro* proliferation in response to 10% FBS (*J*) and viability in cells incubated in serum-free media for 48 h (*K*) were assessed with sh-Control and shGEF17 LAP0297 cells with the MTT assay. Fifteen hundred cells per well were left overnight in 1% FBS for proliferation assays and 50,000 cells for viability experiments, followed by 48 h in media containing 10% FBS (*J*) or in serum-free media (*K*). Proliferation and viability were evaluated with the MTT assay; in both cases, cells incubated in 1% FBS were used as a control. Graphs represent the mean ± SEM. *J*, n = 4: ∗∗∗*p* < 0.001, ∗∗*p* < 0.01, ns; *t* test. *K*, n = 4; ∗*p* < 0.05, ns; *t* test. MTT, 3-(4,5-dimethylthiazol-2-yl)-2,5-diphenyl-2H-tetrazolium bromide; ns, nonsignificant; NSCLC, non–small cell lung carcinoma.

To get an initial insight into the potential role of ARHGEF17 in lung cancer we looked for clinical differences in patients having this gene amplified compared with all others. Analysis of the non–small cell lung carcinoma (NSCLC) TCGA datasets (61) revealed that ARHGEF17 amplification correlated with a higher percentage of patients with tumors at stages II–IV and N1–2, indicative of advanced tumor growth and lymph node infiltration, respectively (https://www.cbioportal.org/; Fig. 1, *A* and *B*; ARHGEF17 is named GEF17 in all the figures). Consistent with these observations, maximally separated Kaplan–Meier survival plots of patients with high-grade tumors (stage II–IV) had a significant statistical correlation between high ARHGEF17 expression and reduced survival (Fig. 1*C*). In contrast, patients with tumors classified as stage I did not show statistical correlation between high ARHGEF17 expression and overall survival (not shown). These analyses were consistent with a hypothetical role of ARHGEF17 in lung cancer progression.

To directly address the potential role of ARHGEF17 in metastatic lung cancer, we knocked down this RhoGEF in LAP0297 lung cancer cells using a lentiviral shRNA-ARHGEF17 (Fig. 1*D*) to address their tumorigenic and organotropic metastatic potential in immunocompetent FVB mice. LAP0297 shGEF17 cells, with reduced expression of ARHGEF17, developed smaller tumors compared with sh-Control cells (Fig. 1*E*), which, at the end of the experiment, weighted significantly less (Fig. 1, *F* and *G*). The metastatic potential of LAP0297-shGEF17 knockdown lung cancer cells, compared with sh-Control cells, was evaluated in FVB mice that were intravenously injected in the tail with 500,000 cells. Metastatic tumors grown in the lungs were evaluated after 2 weeks. The macroscopic appearance of normal lungs and those excised from mice injected with sh-Control and shGEF17 cells is shown in Figure 1*H*. Lungs with multiple metastatic tumors weighted more than normal lungs from healthy mice (Fig. 1*H*). Interestingly, both the weight (Fig. 1*H*) and the number of lung macrometastasis (Fig. 1*I*) were significantly attenuated in mice inoculated with cells in which ARHGEF17 was knocked down in comparison to those inoculated with sh-Control cells. The putative antiproliferative effect of ARHGEF17 knockdown and the potential effect on cell viability were assayed in sh-Control and shGEF17-cultured LAP0297 cells. For these experiments, cells were incubated overnight with 1% fetal bovine serum (FBS) and then incubated with 10% FBS or serum-free media for 48 h and analyzed with the 3-(4,5-dimethylthiazol-2-yl)-2,5-diphenyl-2H-tetrazolium bromide assay. ARHGEF17 knockdown shGEF17 cells were able to proliferate in response to 10% FBS (Fig. 1*J*), and their viability was not significantly reduced in the prolonged absence of serum (Fig. 1*K*). However, when compared with sh-Control cells, the proliferative effect was slightly reduced (Fig. 1*J*) as well as the viability in the continued absence of serum (Fig. 1*K*). Together, these results suggest that ARHGEF17 plays a critical role in lung cancer growth and metastatic dissemination.

### ARHGEF17 is involved in LPA-elicited cell migration and invasion

We previously demonstrated that LPA stimulates metastatic LAP0297 cells to migrate *via* a signaling pathway sensitive to pertussis toxin (PTX) and gallein, indicating the critical participation of heterotrimeric Gi proteins controlling a still to be revealed migratory mechanism based on Gβγ-effector pathways (50). Consistent with the importance of LPA signaling in lung cancer (41), analysis of transcriptomic information from the NSCLC TCGA datasets revealed a positive correlation between ARHGEF17 and autotaxin (ectonucleotide pyrophosphatase/phosphodiesterase 2 [ENPP2]) expression and increased levels of this enzyme, which catalyzes the extracellular production of LPA (41), correlated with reduced survival of advanced cancer patients (Fig. S1).

**Figure 2:**
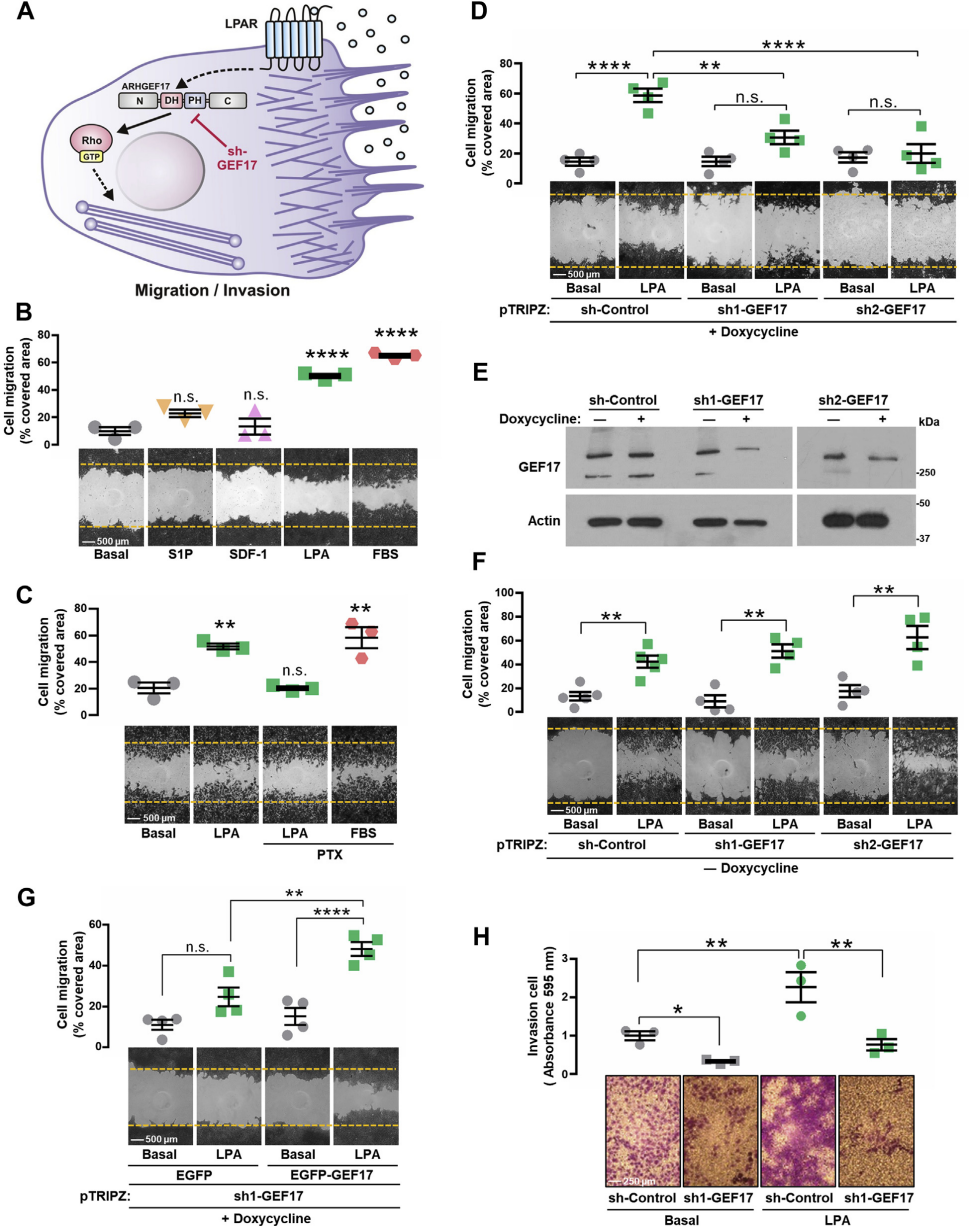
**LPA promotes cell migration and invasion *via* ARHGEF17.***A*, hypothetical model representing ARHGEF17 participation in cell migration/invasion stimulated by LPA. *B*, migration assays of LAP0297 cells stimulated with S1P (1 μM), SDF-1 (10 ng/ml), LPA (5 μM), and FBS as control. Graphs represent the mean ± SEM, n = 3; ∗∗∗∗*p* < 0.0001; ns; one-way ANOVA followed Dunnett's test. The scale represents 500 μm. *C*, LAP0297 cells were simulated with LPA (5 μM) in the presence or absence of 100 ng/ml of PTX. FBS was used as control. Data represent the mean ± SEM, n = 3; ∗∗*p* < 0.01, ns; one-way ANOVA followed Dunnett's test. *D*, LAP0297 cells with the indicated doxycycline-inducible shRNAs were tested on migration assays. Data represent the mean ± SEM, n = 4; ∗∗∗∗*p* < 0.0001, ∗∗*p* < 0.01; one-way ANOVA followed Tukey's test; ns. *E*, expression of GEF17 and actin in sh-Control and shGEF17 cells, grown in the presence or the absence of doxycycline, was detected by Western blot. *F*, migration assay of sh-Control and shGEF17 LAP0297 cells grown in the absence of doxycycline. Graph represents the mean ± SEM, n = 4. ∗∗*p* < 0.01; *t* test. *G*, knockdown LAP0297 cells (sh1-GEF17 knockdown cells) grown in the presence of doxycycline (1 μg/ml) were transfected with EGFP or EGFP-human ARHGEF17 (EGFP-GEF17) and subjected to migration in response to 5 μM LPA. Graph represents the mean ± SEM, n = 4. ∗∗∗∗*p* < 0.0001, ∗∗*p* < 0.01, ns. One-way ANOVA followed by Tukey's test. *H*, invasive ability of control and GEF17-knockdown LAP0297 cells stimulated with LPA was analyzed on Matrigel-coated filters. Data represent the mean ± SEM; n = 3; ∗∗*p* < 0.01, ∗*p* < 0.05; *t* test. The scale represents 250 μm. EGFP, enhanced GFP; FBS, fetal bovine serum; LPA, lysophosphatidic acid; ns, nonsignificant; PTX, pertussis toxin.

Given the reduced tumorigenic and metastatic power of ARHGEF17-knockdown cells (Fig. 1, *E*–*I*), we explored the cellular and molecular mechanisms by which ARHGEF17 might play a critical role in lung cancer. As depicted in Figure 2*A*, we hypothesized that ARHGEF17 plays a major role in cell invasion and migration elicited by LPA. We first compared the response of LAP0297 cells to LPA, S1P, and SDF-1 (CXCL12), agonists of Gi-coupled receptors that have been implicated in cancer cell dissemination (42, 47, 62, 63). We found that only LPA and FBS, used as positive control, had a significant effect on cell migration (Fig. 2*B*). Moreover, consistent with our previous findings (50), PTX inhibited the migratory response of LAP0297 cells to LPA but not to FBS (Fig. 2*C*) or hepatocyte growth factor (Fig. S2), confirming the participation of Gi in the pathway activated by LPA. Cell migration in response to LPA was reduced in cells infected with lentiviral vectors carrying two different doxycyclin-inducible shRNAs that effectively inhibited ARHGEF17 expression (Fig. 2, *D* and *E*), but not in cells grown in the absence of doxycycline (Fig. 2*F*), condition in which the expression of ARHGEF17 was maintained (Fig. 2*E*). Next, to validate the specificity of ARHGEF17 shRNA, we transfected human enhanced GFP (EGFP)-tagged ARHGEF17 into LAP0297 shGEF17 knockdown cells. As shown in Figure 2*G*, overexpression of human ARHGEF17 rescued the ability of mouse shGEF17-knockdown cells to migrate in response to LPA. Furthermore, the ability of LAP0297 cells to invade matrigel-covered filters in response to LPA was significantly reduced by silencing ARHGEF17 (Fig. 2*H*). Altogether, these results revealed the critical role played by ARHGEF17 in the migratory and invasive response of metastatic LAP0297 lung cancer cells to LPA. Mechanistically, this is putatively linked to the activation of RhoA, the small GTPase that, according to *in vitro* assays, is specifically recognized by ARHGEF17 (52).

### ARHGEF17 overexpression increases cell proliferation and migration

**Figure 3:**
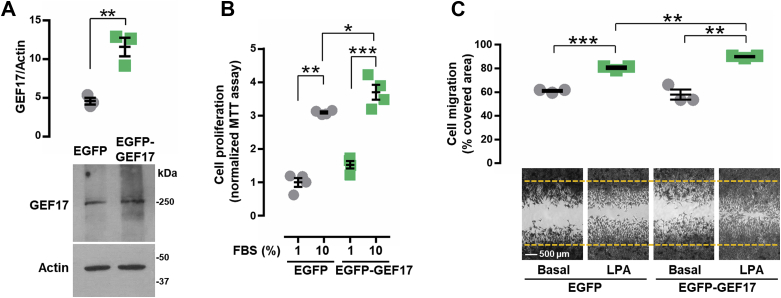
**ARHGEF17 overexpression increases cell proliferation and migration.***A*, LAP0297 cells were transfected with EGFP or EGFP-GEF17, which was revealed by Western blot from with anti-GEF17 antibodies. Graph represents the mean ± SEM; n = 3; ∗∗*p* < 0.01; *t* test. *B*, proliferation assay of LAP0297 cells (EGFP or EGFP-GEF17) stimulated with 10% FBS. Graph represents the mean ± SEM, n = 4. ∗∗∗*p* < 0.001, ∗∗*p* < 0.01, ∗*p* < 0.05; *t* test. *C*, migration assay of LAP0297 cells (EGFP or EGFP-GEF17) stimulated with 5 μM LPA. Graph represents the mean ± SEM; n = 3. ∗∗∗*p* < 0.001, ∗∗*p* < 0.01; *t* test. The scale represents 500 μm. EGFP, enhanced GFP; FBS, fetal bovine serum; LPA, lysophosphatidic acid.

To address the potential effects of ARHGEF17 overexpression on cell proliferation and migration, LAP0297 lung cancer cells were transfected with either EGFP or EGFP-ARHGEF17. Expression of ARHGEF17 was significantly increased in the corresponding transfected cells (Fig. 3*A*). Compared with EGFP-transfected cells, ARHGEF17-overexpressing cells exhibited a slight, but significant, increase on cell proliferation in response to 10% FBS (Fig. 3*B*) and migration in response to 5 μM LPA (Fig. 3*C*).

### LPA activates ARHGEF17

**Figure 4:**
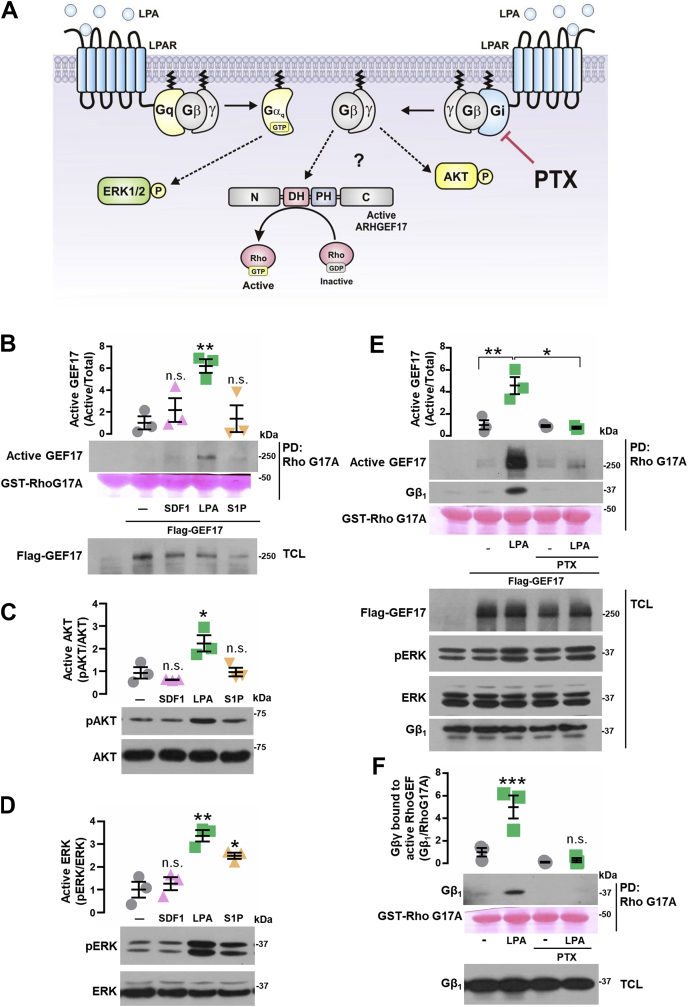
**LPA activates ARHGEF17 *via* Gi.***A*, hypothetical model representing the role of Gi, assessed by pertussis toxin (PTX), on ARHGEF17 activation by LPA. *B*, active GEF17 was isolated by RhoG17A pull down from FLAG-GEF17-transfected LAP0297 cells stimulated with SDF-1 (50 ng/ml), LPA (5 μM), or S1P (1 μM) for 15 min. Graph represents the mean ± SEM, n = 3; ∗∗*p* < 0.01, one-way ANOVA followed Dunnett's test; ns. *C* and *D*, phosphorylation of AKT (*C*, pAKT) and ERK (*D*, pERK) was analyzed by Western blot with lysates from LAP0297 cells stimulated as indicated in (*B*). *C*, pAKT: ∗*p* < 0.05, one-way ANOVA followed Dunnett's test; ns. *D*, pERK: ∗∗*p* < 0.01, one-way ANOVA followed Dunnett's test; ns. *E*, active GEF17 was isolated by RhoG17A pull down from LAP0297 cells expressing FLAG-GEF17. Cells were incubated overnight in the presence or the absence of 100 ng/ml of PTX, then stimulated with LPA (5 μM). Data represent the mean ± SEM, n = 3; ∗*p* < 0.05, ∗∗*p* < 0.01; two-way ANOVA followed Tukey's test. A representative blot is shown below the graph. *F*, Gβ_1_ was detected in the active GEF17 pull-down assays shown in (*E*). ∗∗∗*p* < 0.001, ns, one-way ANOVA followed Dunnett's test. ERK, extracellular-regulated kinase; LPA, lysophosphatidic acid; ns, nonsignificant.

Given that ARHGEF17 was proven to be essential for the migratory and invasive responses elicited by LPA in metastatic lung cancer cells (Fig. 2, *D*–*H*), and our previous studies demonstrating that heterotrimeric Gi proteins participate in the migratory response to this agonist (50); we hypothesized that Gi activates ARHGEF17 *via* Gβγ (Fig. 4*A*). We directly addressed whether ARHGEF17 was selectively activated by LPA. Using recombinant RhoA-G17A as an affinity matrix to isolate the active fraction of ARHGEF17 (64), we found that LPA, but not SDF-1 or S1P, selectively activated this RhoGEF (Fig. 4*B*). In addition, LPA stimulated AKT (Fig. 4*C*) and extracellular-regulated kinase (ERK) (Fig. 4*D*). In contrast, S1P only had a significant effect on ERK, whereas SDF-1 was unable to stimulate these signaling effectors (Fig. 4, *C* and *D*). Furthermore, activation of ARHGEF17 in response to LPA was inhibited by PTX, indicating that this RhoGEF is an effector of Gi (Fig. 4*E*). Since Gβγ released from Gi has been reported as essential to promote Gi-dependent cell migration by GPCRs, and this heterodimer directly drives the activation of RhoGEFs such as P-Rex1 (65, 66), we inferred the presence of Gβγ in the active fraction of ARHGEF17. We identified endogenous Gβ1 bound to the active fraction of ARHGEF17 in control cells treated with LPA but not in cells in which agonist-stimulated dissociation of Gi heterotrimer was inhibited by PTX (Fig. 4, *E* and *F*). Treatment with PTX did not inhibit ERK activation by LPA (Fig. 4*E*; phospho-ERK); indicating that LPA activates Gi-dependent and Gi-independent pathways in LAP0297 cells. Altogether, these results show that LPA signaling activates ARHGEF17 in a Gi-dependent manner involving potential direct interactions with Gβγ.

### Gβγ binds ARHGEF17 promoting its localization at the cell periphery

**Figure 5:**
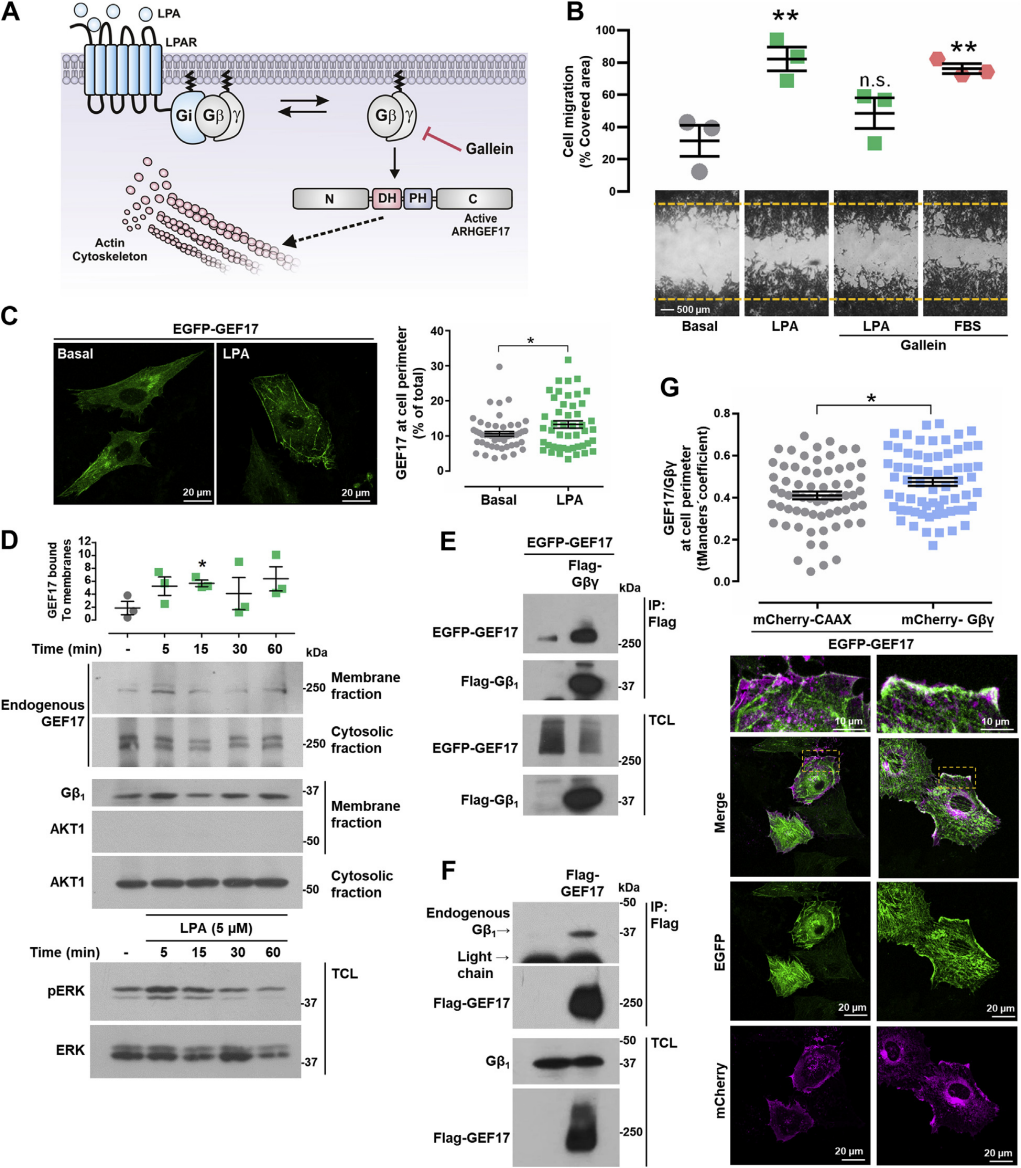
**Gβγ recruits ARHGEF17.***A*, hypothetical model representing the role of Gβγ (tested with gallein) on ARHGEF17 localization and activity. *B*, migration of LAP0297 cells preincubated or not with gallein (10 μM) was stimulated with LPA (5 μM), FBS served as control. Data represent the mean ± SEM, n = 3; ∗∗*p* < 0.01, one-way ANOVA followed Dunnett's test; ns. *C*, PAE cells were transfected with EGFP-ARHGEF17 and stimulated with LPA (5 μM) for 30 min, fixed and visualized by confocal microscopy. Graph represents the mean ± SEM of GEF17 detected at the cell perimeter (percent of total, 50, and 47 cells, in basal or stimulated conditions, respectively, was analyzed with FIJI-ImageJ software). ∗*p* < 0.05; *t* test. The scale represents 20 μm. *D*, LAP0297 cells were stimulated with LPA (5 μM) for the indicated times, and membrane and cytosolic fractions were obtained as described in the Experimental procedures section and analyzed by Western blot to detect the fraction of ARHGEF17 recruited to the membrane. Heterodimeric Gβγ, revealed by Western blot against Gβ_1_, served as membrane marker and for normalization of ARHGEF17 bound to the membrane fraction. AKT1 served as cytosolic marker. Cell stimulation was confirmed by detecting the phosphorylation of ERK (pERK). A representative blot from three independent experiments is shown. Graph represents the mean ± SEM. n = 3. ∗*p* < 0.05; *t* test. *E*, coimmunoprecipitation of GEF17 and Gβγ was analyzed using lysates from HEK 293T cells transfected with EGFP-GEF17 together or not with FLAG-Gβγ. Immunoprecipitation was done with FLAG antibodies. GEF17 and Gβ_1_ were detected by Western blot. A representative blot from three independent experiments is shown. *F*, FLAG-GEF17 was immunoprecipitated from transfected HEK 293T cells, and interacting endogenous Gβγ was detected by Western blot with antibodies against Gβ_1_. Western blot is representative of three independent experiments. *G*, effect of Gβγ on ARHGEF17 localization was analyzed by confocal fluorescence microscopy of PAE cells expressing EGFP-GEF17 with mCherry-Gβ_1_γ_2_ or mCherry-CAAX, as control. Graph represents the *t* Mander's coefficient of colocalization of EGFP-ARHGEF17 with mCherry-Gβγ (66 cells per condition, from three independent experiments, were analyzed with the Coloc2 plugin of the FIJI-ImageJ software). Graph represents the mean ± SEM. ∗*p* < 0.05; *t* test. Representative cells are shown below the graph. The scale represents 20 μm; zoom represents 10 μm. EGFP, enhanced GFP; ERK, extracellular-regulated kinase; FBS, fetal bovine serum; HEK 293T, human embryonic kidney 293T cell line; LPA, lysophosphatidic acid; ns, nonsignificant; PAE, porcine aortic endothelial.

The presence of Gβγ in the active fraction of ARHGEF17 (Fig. 4, *E* and *F*) supported a potential mechanistic link between these signaling proteins relevant to drive actin cytoskeleton reorganization and cell migration in lung cancer cells responding to LPA (Fig. 5*A*). To start addressing this possibility, we first evaluated the participation of Gβγ in the migratory response of LAP0297 cells. Inhibition of Gβγ with gallein (67) interfered with the migratory response of LAP0297 cells to LPA but not to FBS used as control (Fig. 5*B*). We hypothesized that, to be activated by LPARs, ARHGEF17 has to be recruited to the plasma membrane where the interaction with Gβγ would be mechanistically relevant. Then, using porcine aortic endothelial (PAE) cells transfected with EGFP-ARHGEF17, we assessed the effect of LPA on ARHGEF17 subcellular localization, visualized by confocal microscopy. Consistent with the activation of LPA signaling pathway that promotes ARHGEF17 recruitment to the plasma membrane, we found that the amount of ARHGEF17 at the cell perimeter in cells stimulated with 5 μM LPA was significantly higher compared with the basal condition (Fig. 5*C*). Consistently, endogenous ARHGEF17 was recruited, in a time course–dependent manner, to the membrane fraction of LAP0297 cells stimulated with 5 μM LPA, showing a significant increase at 15 min of stimulation (Fig. 5*D*). The same membrane fraction had Gβγ (revealed by Western blot anti-Gβ1), which was used to normalize the amount of ARHGEF17 in the membrane fraction. To confirm that the membrane fraction was not contaminated with cytosolic proteins, we looked for the presence of AKT1 in the membrane and cytosolic fractions, detecting by Western blot the presence of this kinase only in the cytosolic fraction (Fig. 5*D*). Erk phosphorylation and total ERK were revealed in total cell lysates to confirm proper cell stimulation with LPA. With coimmunoprecipitation assays, we detected interaction between FLAG-Gβγ and EGFP-ARHGEF17 (Fig. 5*E*). In addition, endogenous Gβγ coimmunoprecipitated with FLAG-ARHGEF17 (Fig. 5*F*). Then, we evaluated whether Gβγ contributes to the recruitment of ARHGEF17 at the cell periphery. EGFP-ARHGEF17 and mCherry-Gβγ (with the mCherry tag fused to the Gγ subunit) were expressed in PAE endothelial cells, and transfected cells were analyzed by confocal microscopy. When expressed with mCherry-CAAX, most EGFP-ARHGEF17 exhibited a cytosolic localization (Fig. 5*G*, *left panels*); in contrast, cells coexpressing mCherry-Gβγ and EGFP-ARHGEF17 had a significant fraction of the RhoGEF colocalizing with Gβγ (Fig. 5*G*, *right panels* and *graph*). Regarding the perinuclear localization of a fraction of Gβγ (Fig. 5*G*, *bottom right panel*), the signal might represent newly synthesized heterodimers, which have been reported to be trafficking *via* the perinuclear endoplasmic reticulum upon assembly and isoprenylation of Gγ (68, 69). Thus, LPA stimulates cell migration *via* Gβγ, which recruits ARHGEF17 to the plasma membrane, putatively driving its activation.

### Gβγ promotes actin cytoskeleton reorganization *via* ARHGEF17

**Figure 6:**
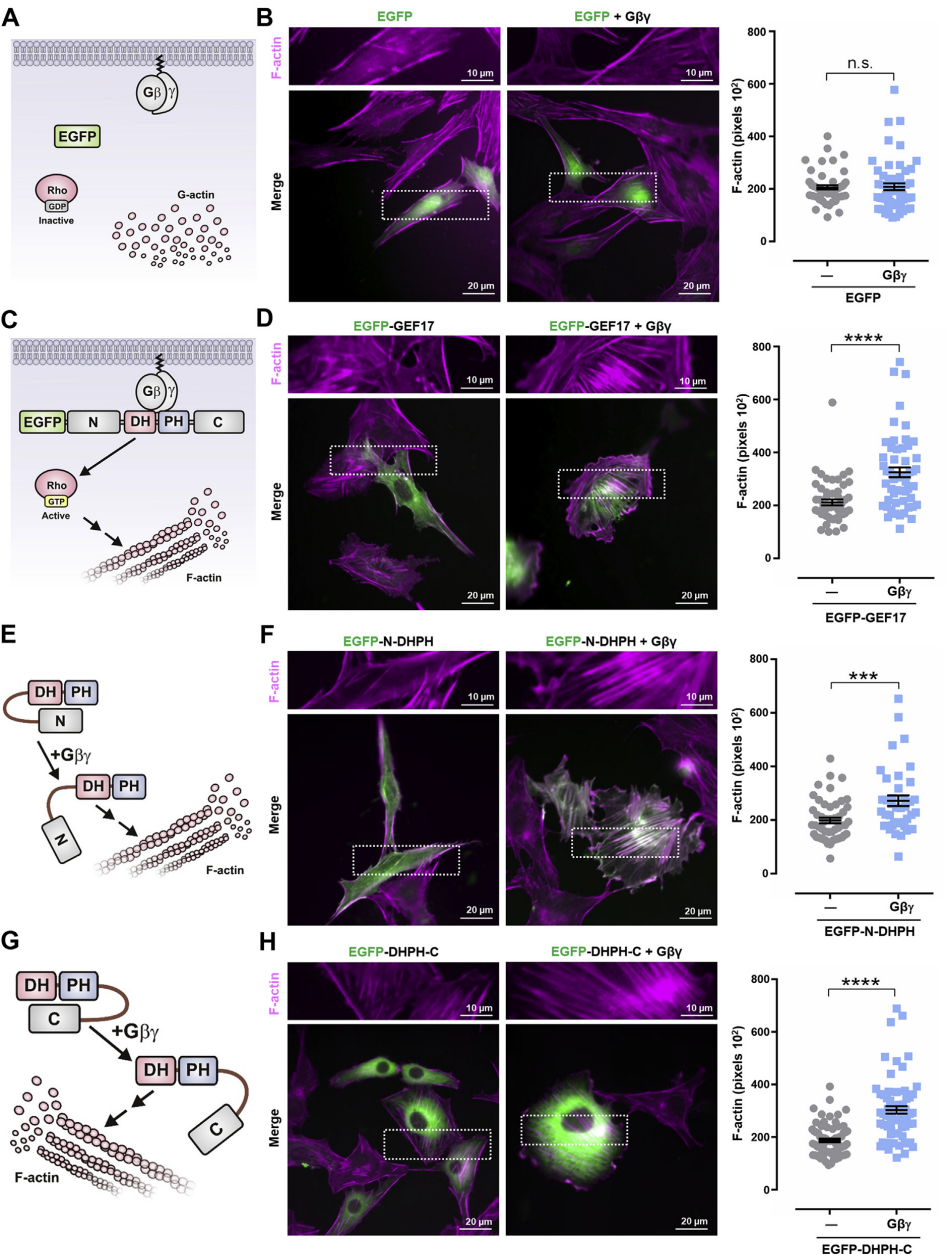
**Gβγ drives ARHGEF17-dependent actin cytoskeleton reorganization.** The hypothetical effect of Gβγ on ARHGEF17-dependent actin cytoskeleton reorganization is depicted in (*C*, *E*, and *G*). Control conditions are depicted in (*A*). Representative PAE cells stained with red fluorescent phalloidin are shown in (*B*, *D*, *F*, and *H*). Cells were transfected with EGFP, EGFP-GEF17, EGFP-GEF17-N-DHPH, EGFP-GEF17-DHPH-C together or not with FLAG-Gβγ as indicated. Graphs represent the mean ± SEM from three independent experiments (at least 30 cells per condition). ∗∗∗∗*p* < 0.0001, ∗∗∗*p* < 0.001; *t* test; ns. The scale represents 20 μm; zoom represents 10 μm. EGFP, enhanced GFP; ns, nonsignificant; PAE, porcine aortic endothelial.

Since ARHGEF17 is recruited by Gβγ, we hypothesized that this signaling heterodimer could promote actin cytoskeleton reorganization *via* this RhoGEF. To test this possibility, we used PAE cells transfected with either full-length ARHGEF17 or constructs containing the DH–PH catalytic module extended toward the N-terminal or C-terminal regions, alone or in combination with Gβγ (Fig. 6, *A*, *C*, *E*, and *G*). These constructs were tagged with EGFP, and cells were stained with fluorescent phalloidin to visualize the organization of actin fibers. Control cells transfected with EGFP with or without Gβγ did not show an increase in the amount of filamentous actin (Fig. 6*B*). As predicted, coexpression of Gβγ with full-length EGFP-ARHGEF17 (Fig. 6*D*), EGFP-N-DHPH (Fig. 6*F*), and EGFP-DHPH-C constructs (Fig. 6*H*) led to an increase on actin fibers, indicating that Gβγ might target the DH–PH module, stimulating its activity on RhoA. Regarding its localization, in cells cotransfected with Gβγ, full-length EGFP-ARHGEF17 (Fig. 6*D*, *right picture*, *merge*) and the EGFP-N-DHPH construct (Fig. 6*F*, *right picture*, *merge*), but not the EGFP-DHPH-C (Fig. 6*H*, *right picture*, *merge*), localized to actin fibers (observed as *white fibers* in the colocalization), suggesting that the previously identified actin-binding motif, found at the N-terminal region (59), is exposed in the presence of Gβγ.

### ARHGEF17 maintains inhibitory intramolecular interactions

**Figure 7:**
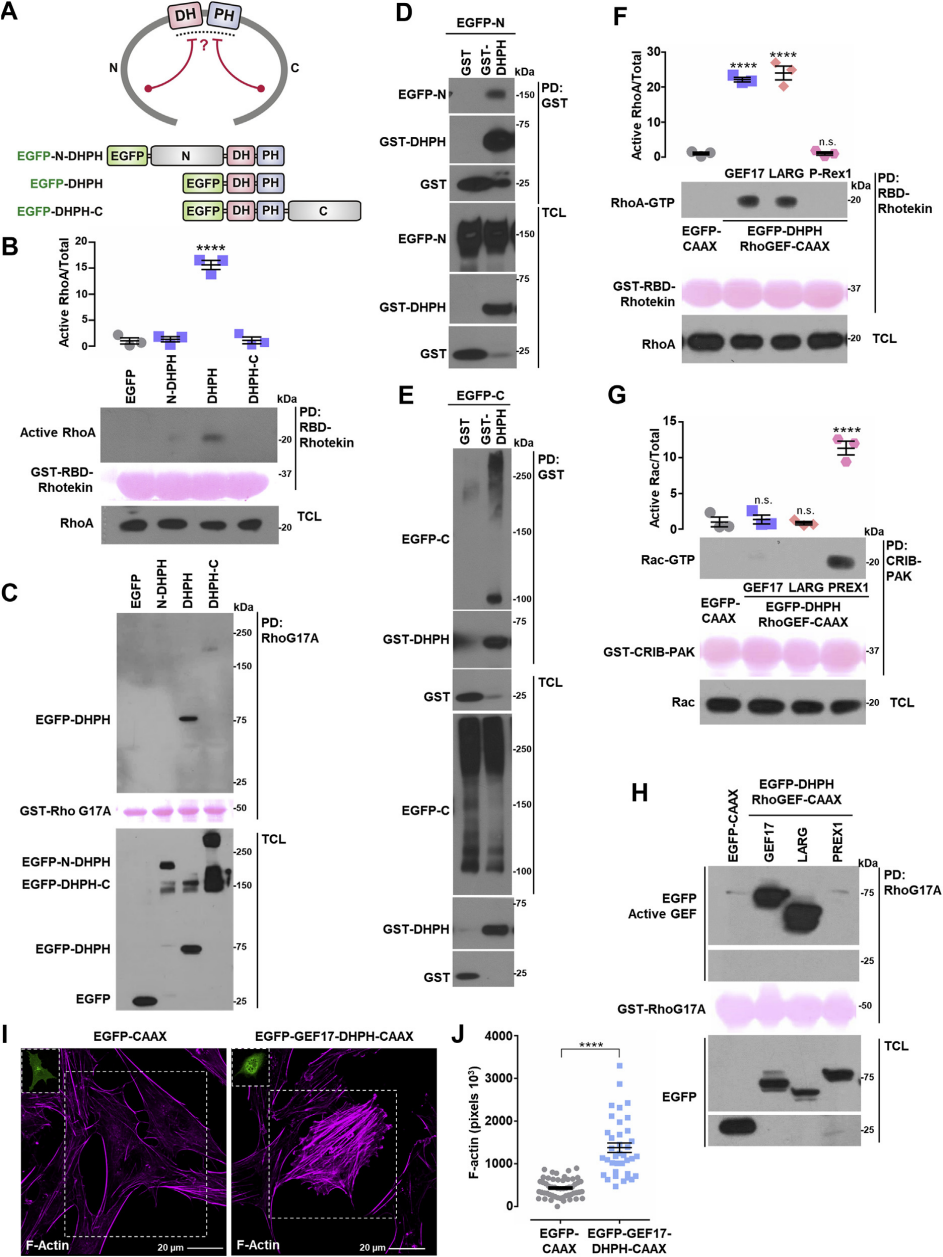
**ARHGEF17 intramolecular interactions.***A*, hypothetical model of GEF17 inhibitory intramolecular interactions and the constructs used to address the possibility. *B* and *C*, active RhoA (*B*) and active RhoGEF (*C*) were isolated by pull down from HEK 293T cells expressing EGFP, EGFP-GEF17-N-DHPH, EGFP-GEF17-DHPH, or EGFP-GEF17-DHPH-C. *B*, graph represents the mean ± SEM, n = 3. ∗∗∗∗*p* < 0.0001; one-way ANOVA followed Dunnett's test. A representative blot is shown in (*C*). *D* and *E*, GST-GEF17-DHPH was isolated by pull down from HEK 293T cells also expressing either EGFP-tagged GEF17-N (*D*) or GEF17-C (*E*) domains. GST was used as control. EGFP- and GST-tagged proteins were revealed by Western blot in pull downs and total cell lysates. Blots are representative of three independent experiments. *F* and *G*, active RhoA (*F*) and Rac (*G*) were isolated from HEK 293T cells transfected with EGFP-DHPH-RhoGEF-CAAX constructs (GEF17, LARG, and P-Rex1). *F*, graph represents the mean ± SEM, n = 3. ∗∗∗∗*p* < 0.0001, ns, one-way ANOVA followed Dunnett's test. *G*, graph represents the mean ± SEM, n = 3. ∗∗∗∗*p* < 0.0001, ns, one-way ANOVA followed Dunnett's test. *H*, active RhoGEFs were isolated by pull down with GST-RhoG17A from HEK 293T cells transfected with EGFP-DHPH-RhoGEF-CAAX constructs (GEF17, LARG, and P-Rex1). A representative Western blot is shown. *I* and *J*, effect of EGFP-GEF17-DHPH-CAAX on the actin cytoskeleton of transfected PAE cells stained with red fluorescent phalloidin. Representative cells are shown in (*I*), control cells were transfected with EGFP-CAAX. The scale represents 20 μm. *J*, graph represents the mean ± SEM, EGFP-CAAX (54 cells), and EGFP-GEF17-DHPH-CAAX (37 cells). ∗∗∗∗*p* < 0.0001; *t* test. EGFP, enhanced GFP; GST, glutathione-*S*-transferase; HEK 293T, human embryonic kidney 293T cell line; ns, nonsignificant; RhoGEF, Rho guanine nucleotide exchange factor.

To get further insight into the molecular mechanism of ARHGEF17 regulation, we first evaluated the possibility that this RhoGEF was autoinhibited by intramolecular interactions (Fig. 7*A*) (52). This possibility was further supported by the lack of effect that ARHGEF17 constructs had in the absence of Gβγ on the actin cytoskeleton of endothelial cells (Fig. 6, *D*, *F*, and *H*, compared with Fig. 6*B*, *left upper pictures*). We directly addressed whether EGFP-tagged ARHGEF17 constructs containing only the catalytic DH–PH module or extending toward the N-terminal or C-terminal domains were active, and whether the N-terminal and C-terminal regions interacted with the DH–PH module. Consistent with the notion of inhibitory intramolecular interactions, only the DH–PH construct, but not those extending from this catalytic region toward the N-terminal or C-terminal regions, stimulated RhoA (Fig. 7*B*). Importantly, the DH–PH construct was the only one with GEF activity, as judged by its binding to nucleotide-free RhoA (Fig. 7*C*). Furthermore, the DH–PH module, expressed as a glutathione-*S*-transferase (GST)-tagged construct, interacted with both the N-terminal and C-terminal domains of ARHGEF17 (Fig. 7, *D* and *E*). In total cell lysates, ARHGEF17-C (EGFP-C) was detected as a 100 kDa band and a high molecular smear (Fig. 7*E*, *bottom panel*), whereas the fraction of ARHGEF17-C preferentially pulled down with the GST-GEF17-DHPH construct was the 100 kDa GEF17-C band (Fig. 7*E*, *upper panel*). Similarly, the ARHGEF17-DHPH-C construct was detected as two bands, one of 150 kDa and the other above 250 kDa (Fig. 7*C*, *bottom panel*, *last lane*). These results might indicate that ARHGEF17-C domain interacts either intramolecularly with the DH–PH domains or intermolecularly with the ARHGEF17-C domain of other ARHGEF17 molecule. To further characterize the activity and specificity of ARHGEF17-DHPH, we prepared an EGFP-tagged construct anchored to the membrane by introducing a CAAX isoprenylation motif. This construct, predicted to be constitutively active, was assayed together with LARG and P-Rex1 DH–PH constructs, known to activate Rho and Rac, respectively (70, 71). ARHGEF17, LARG, and P-Rex1 EGFP-DHPH-CAAX constructs maintained their specificity (Fig. 7, *F* and *G*). As predicted, ARHGEF17-DHPH-CAAX and LARG-DHPH-CAAX activated Rho but not Rac (Fig. 7, *F* and *G*), whereas P-Rex1-DHPH-CAAX was active on Rac but not on Rho (Fig. 7, *F* and *G*). The specificity of the active RhoGEF pull-down assay was also confirmed, as indicated by the experiments with recombinant GST-RhoG17A, which specifically pulled down active ARHGEF17-DHPH-CAAX and LARG-DHPH-CAAX but not the P-Rex1 construct (Fig. 7*H*). The constitutive activity of ARHGEF17-DHPH-CAAX was further demonstrated in PAE cells transfected with this construct, which caused a significant increase on actin polymerization, compared with EGFP-CAAX–transfected cells (Fig. 7, *I* and *J*). Together, these results support the existence of a self-inhibiting mechanism where the catalytic domain is blocked by interactions with the amino and carboxyl terminal domains.

### Gβγ interacts with ARHGEF17 PH and C-terminal domains

**Figure 8:**
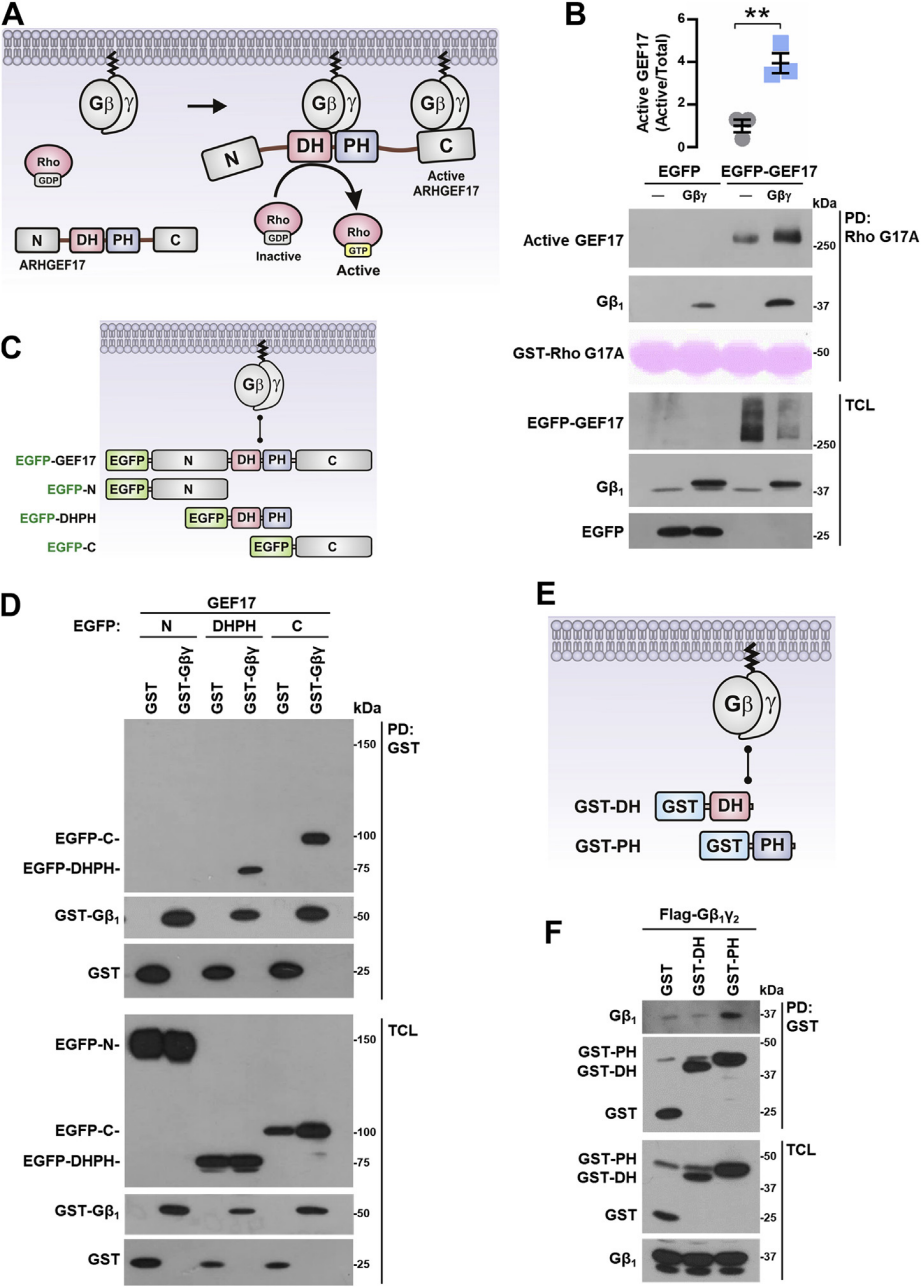
**Gβγ activates ARHGEF17 to which it binds at the PH and C domains.***A*, hypothetical model representing GEF17 activation by Gβγ. *B*, active GEF17 was isolated by pull down with GST-RhoG17A from HEK 293T cells cotransfected with Gβγ or control plasmid. Graph represents the mean ± SEM; n = 3; ∗∗*p* < 0.01; *t* test. *C*, model depicts potential interaction between Gβγ and GEF17 and the constructs used to address it. *D*, GST-Gβγ or GST (as control) was isolated by pull down from HEK 293T cells cotransfected with EGFP-tagged GEF17 constructs: EGFP-GEF17-N, EGFP-GEF17-DHPH, or EGFP-GEF17-C. EGFP-tagged and GST-tagged proteins were revealed by Western blot in pull downs and total cell lysates. Blot is representative of three independent experiments. *E*, potential interaction of Gβγ with GEF17 DH and PH domains and the constructs used to test this possibility. *F*, GST-GEF17-DH and GST-GEF17-PH were isolated by pull down from HEK 293T cells cotransfected with FLAG-Gβγ. Proteins were detected by Western blot in pull downs and total cell lysates. Blot is representative of three independent experiments. EGFP, enhanced GFP; GST, glutathione-*S*-transferase; HEK 293T, human embryonic kidney 293T cell line.

To further characterize the functional consequences of Gβγ–ARHGEF17 interaction, demonstrated by immunoprecipitation (Fig. 5, *E* and *F*), we tested the putative activation of this RhoGEF by its interaction with Gβγ (Fig. 8*A*). Pull-down assays to capture active RhoGEFs with recombinant RhoA-G17A revealed that Gβγ stimulated EGFP-tagged full-length ARHGEF17 (Fig. 8*B*). We then mapped the Gβγ-binding interfaces on ARHGEF17 using EGFP-tagged N, DH–PH, and C constructs (Fig. 8*C*). GST-Gβγ, or GST as control, was cotransfected with these ARHGEF17 constructs and subjected to pull-down assays. Consistent with the existence of two independent interaction contacts (Fig. 8*A*), Gβγ bound the DH–PH and C-terminal modules of ARHGEF17 but not the construct corresponding to the N-terminal domain (Fig. 8*D*). When the DH and PH domains were expressed as independent entities (Fig. 8*E*), Gβγ interacted with the PH but not the DH domain (Fig. 8*F*). Altogether, these results indicate that Gβγ interacts on two independent regions of ARHGEF17, promoting its activation.

### Gβγ allosterically activates ARHGEF17 by displacing intramolecular inhibitory interactions

**Figure 9:**
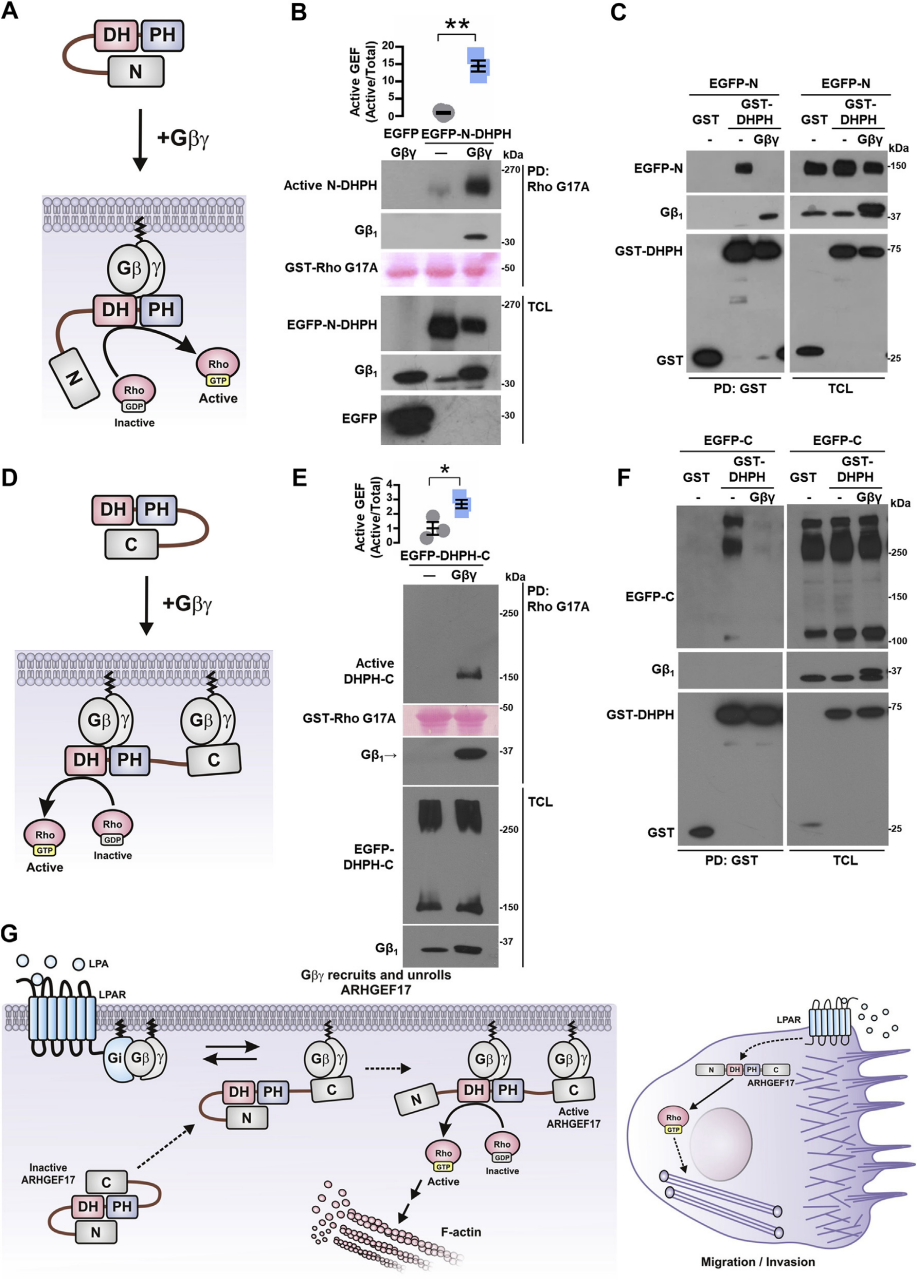
**Gβγ activates ARHGEF17 by displacing intramolecular interactions.***A*, hypothetical model representing how Gβγ activates the GEF17-N-DHPH construct. *B*, active GEF17-N-DHPH was isolated from HEK 293T cells cotransfected or not with Gβγ. Graph represents the mean ± SEM from three independent experiments. ∗∗*p* < 0.01; *t* test. *C*, GST-GEF17-DHPH or GST (as control) was isolated by pull down from HEK 293T cotransfected with GFP-GEF17-N domain (EGFP-N) in the presence or the absence of Gβγ. Transfected proteins were detected by Western blot in pull downs and total cell lysates. Blot is representative of four independent experiments. *D*, hypothetical model representing how Gβγ activates the GEF17-DHPH-C construct. *E*, active GEF17-DHPH-C was isolated from HEK 293T cells cotransfected with EGFP-GEF17-DHPH-C construct in the presence or the absence of Gβγ. Graph represents the mean ± SEM from three independent experiments. ∗*p* < 0.05; *t* test. *F*, GST-GEF17-DHPH or GST (as control) was isolated by pull down from HEK 293T cells cotransfected with EGFP-GEF17-C domain (EGFP-C) in the presence or the absence of Gβγ. Transfected proteins were detected by Western blot in pull downs and total cell lysates. Blot is representative of four independent experiments. *G*, model depicting how ARHGEF17 is recruited and activated by Gβγ. Gi-coupled receptors release Gβγ from Gi. Gβγ recruits ARHGEF17 to the plasma membrane by interaction with the C-terminal domain of the RhoGEF, disrupting an inhibitory interaction that hides the DH–PH domains. A second Gβγ heterodimer interacts with GEF17-DH-PH domains promoting dissociation of GEF17 N-terminal domain, unleashing RhoGEF activity and exposing an actin-binding site at the N-terminal domain contributing to localize GEF17 to the actin cytoskeleton. These dynamic interactions coordinate the spatiotemporal activation of GEF17 during cell migration and invasion. EGFP, enhanced GFP; GST, glutathione-*S*-transferase; HEK 293T, human embryonic kidney 293T cell line; RhoGEF, Rho guanine nucleotide exchange factor.

The finding that Gβγ was essential to promote actin cytoskeleton reorganization by ARHGEF17 N-DHPH (Fig. 6*F*) and DHPH-C constructs (Fig. 6*H*) suggested that the intramolecular restrictions that kept these constructs inactive were removed by the signaling heterodimer (Fig. 9, *A* and *D*). Consistent with this idea, Gβγ stimulated the ARHGEF17 N-DHPH construct and remained bound to it in the RhoA-G17A pull-down assay (Fig. 9*B*). Furthermore, the interaction between ARHGEF17 constructs, GST-DHPH with the EGFP-tagged N-terminal domain, revealed by GST pull down, was removed by Gβγ (Fig. 9*C*, *left panel*). Similarly, Gβγ stimulated the EGFP-tagged ARHGEF17 DHPH-C construct (Fig. 9*E*) and displaced the interaction between the EGFP-C-terminal domain and the DHPH tandem, as revealed by the GST pull down of the catalytic module (Fig. 9*F*, *left panel*). In this case, Gβγ did not remain bound to the GST-DHPH construct, suggesting that it remained bound to the ARHGEF17 C-terminal domain.

## Discussion

The paramount role played by Rho GTPases in the invasive and migratory behavior of cancer and stromal cells within the tumor microenvironment has raised a growing interest on RhoGEFs as crucial effectors and potential therapeutic targets in metastatic cancer (6, 9, 11, 17, 20, 21, 25, 30, 31, 72, 73, 74, 75, 76). Here, we demonstrate that ARHGEF17, component of a transcriptional signature predictive for reduced survival in various cancers (58), is involved in invasion and migration of lung cancer cells elicited by Gi-coupled LPARs. We focused on ARHGEF17 given its original identification as a tumor endothelial marker (51) and target of the Hippo pathway (58). Such expression profile places ARHGEF17 as a potential oncogenic effector. However, no previous studies have addressed this possibility. Consistent with a potential role in human neoplasia, our analysis of the NSCLC TCGA datasets indicated that more patients with stage II–IV tumors had amplified ARHGEF17 (61). In addition, expression of ARHGEF17 and ENPP2 correlated with reduced survival of these patients. Herein, using immunocompetent mice and a syngeneic lung cancer cell line, we demonstrate that ARHGEF17 is indeed involved in tumor growth and metastasis and revealed how Gβγ activates this RhoGEF (depicted in Fig. 9*G*).

Mechanistic ally, LPA activates Gi-coupled receptors and releases Gβγ, which recruits ARHGEF17 to the cell membrane. Gβγ interacts with ARHGEF17 C-terminal and PH domains, releasing intramolecular interactions that, in unstimulated cells, maintain cytosolic ARHGEF17 autoinhibited. To unleash ARHGEF17 catalytic power, Gβγ allosterically uncovers the DH domain, enabling the access of RhoA, its cognate GTPase (52, 59). In addition, it exposes an actin-binding site, present at the N-terminal domain of ARHGEF17 (59). This mechanism likely promotes dynamic adjustments of the actin cytoskeleton that might play a reciprocal regulatory effect on ARHGEF17 activity. This possibility is in agreement with the functional characteristics of a previously described ARHGEF17 mutant, unable to bind polymerized actin (59). Our findings, together with the recent discovery that the actin cytoskeleton controls LPAR1 trafficking in response to self-generated gradient of LPA to sustain directional migration of pancreatic cancer cells (42), supports the general idea that the Gβγ–ARHGEF17 signaling complex might drive polymerization of the actin cytoskeleton as a signaling circuit to control lung cancer cell migration and invasion.

Given that chemotactic GPCRs are well-recognized promoters of tumor growth and dissemination (1, 3, 6, 63, 77), our findings place ARHGEF17 within the repertoire of relevant signaling effectors in oncogenic settings. This Gβγ-regulated RhoGEF joins the canonical repertoire of RGS-RhoGEFs and other transducers of LPARs in diverse cancer cells (7, 34, 38, 39, 42, 48, 78, 79). In addition, catalytic-independent functions of ARHGEF17, including its ability to control a mitotic checkpoint (60), might also play a role in tumor growth, which deserves further investigation. The only other Gβγ-regulated RhoGEFs known to be linked to cancer progression are the two members of the P-Rex1 family (21, 23, 24, 62, 80). Given the contrasting specificity of ARHGEF17 and P-Rex1/2, an emerging possibility is that Gβγ contributes to fine-tune spatiotemporal activation of RhoA and Rac to maintain directional migration (81); a possibility to be considered in future studies.

In conclusion, ARHGEF17 plays an important role in tumor growth and dissemination, which together with the reduced survival of lung cancer patients with advanced disease and high ARHGEF17 expression, are consistent with a pathological role of ARHGEF17 in metastatic cancer. Our results highlight ARHGEF17 as a relevant effector and potential target for therapeutic strategies in metastatic cancer. Given the central role played by Rho GTPases at various stages of tumor progression, our studies set the ground for future investigations aiming to address whether ARHGEF17 is a common effector of Gβγ-dependent signaling pathways elicited by chemotactic GPCRs within the tumor microenvironment.

## Experimental procedures

### Reagents

LPA (catalog no.: 62215) was from Cayman Chemical; SDF-1 (catalog no.: 300-28A) from PeproTech; S1P (catalog no.: S9666), protein-G agarose (catalog no.: 16-266), chemiluminescent substrate (catalog no.:WBKLS0500) and polyvinylidene fluoride membranes (catalog no.: IPVH00010) from Merck. Polyethyleneimine (catalog no.: 23966) was from Polysciences; Polyfect (catalog no.: 301105) from QIAGEN; TurboFect (catalog no.: R053), Lipofectamine 2000 (catalog no.: 11668027), and phalloidin (catalog no.: A12381) from Thermo Scientific; and Glutathione Sepharose (catalog no.: 17-0756-05) from Bio-Sciences AB. Antibiotics for cell culture (catalog no.: 15240062) were from Gibco. Antibodies were from the following sources: Merck (FLAG, F3165; Akt, P2482); Santa Cruz Biotechnology (GFP, sc-9996; GST sc-138; pAkt, sc-7985-R; phosphor-ERK, sc-9101; ERK, sc-154; RhoA, sc-418; Gβ1, sc-261); ProSci (ARHGEF17, 4367); mouse monoclonal antiactin antibody was kindly provided by Dr Manuel Hernandez (Department of Cell Biology, Cinvestav); KPL (antimouse, 074-1802; and anti-rabbit, 074-1516).

### DNA constructs

Full-length pCEFL-3XFLAG-ARHGEF17, obtained from FLJ90019 and KIAA0337 complementary DNA clones, was subcloned as pCEFL-EGFP-ARHGEF17 and served as template for pCEFL-EGFP-ARHGEF17-N-DHPH, pCEFL-EGFP-DHPH-C, and pCEFL2-GST constructs, cloned with the following primers:

hGEF17-aminoM1-5′-NheI ataGCTAGCATGGCGGACGGGGCACCCCGG, hGEF17_amino-(Cys-1059)-3′-EcoRI: ataGAATTCGCAGCACTTGCTGGCAGGGGT; hGEF17_DH(S1060)5′NheI: ataGCTAGCAGCAAGCCACAGGTGGACATGCG, hGEF17_PH(S1476)3′EcoRI: ataGAATTCGCTGGATGCCAGCTTCCTCTTGG; hGEF17_DH3′EcoRI: ataGAATTCCCGCACACCCTTGTTGATGCG

hGEF17_PH5′BamHI: ataGGATCCAGTGCCGAGGAGGCGGAGCGC; hGEF17_COOH(Lys-1477).5′NheI: ataGCTAGCAAAAGCTGTCTAGACCCTGAG

hGEF17_COOH(Val-2063)-3′-EcoRI ataGAATTCCACCCTCCACAGGAGGAGGTG. Other constructs have been previously described (19, 70, 71, 82).

### Cell culture and transfection

LAP0297 kindly provided by Dr Peigen Huang, Harvard Medical School (83), human embryonic kidney 293T (HEK 293T) and PAE cells were routinely grown in Dulbecco's modified Eagle's medium (DMEM) (Invitrogen; catalog no.: 15240112) supplemented with 10% FBS and 1% antibiotic. HEK 293T cells grown on poly-d-lysine–coated plates were transfected with the polyethyleneimine. PAE and LAP0297 cells were transfected with Polyfect and TurboFect, respectively. For cell proliferation and GEF17 rescue experiments, LAP0297 cells were transfected in suspension with Lipofectamine LTX and PLUS reagents (Invitrogen). Experiments were done 48 h post transfection with cells starved in serum-free DMEM, prior to stimulation.

### Active-RhoGEF and Rho GTPase pull-down assays

Active ARHGEF17 was isolated by pull down using GST-RhoG17A beads (64, 71). Briefly, serum-starved confluent HEK 293T or LAP0297 cells were stimulated as indicated in legends to the figures and lysed with 500 μl TBS-Triton (50 mM Tris, pH 7.5, 150 mM NaCl, 1% Triton X-100; without MgCl_2_, and protease inhibitors: 1 mM PMSF, 10 μg/ml leupeptin, 10 μg/ml aprotinin; and phosphatase inhibitors: 10 mM β-glycerophosphate, 1 mM NaF, and 1 mM sodium orthovanadate). A fraction of cell lysate was separated for reference, and the rest was incubated with 35 μl of RhoG17A beads at 4 °C in constant shaking for 45 min. Beads were washed three times with 1 ml of lysis buffer, resuspended with 35 μl of 1× Laemmli sample buffer, and analyzed by Western blot together with total lysates. Active RhoA was isolated by pull-down assays by using GST-RBD-rhotekin beads (71). Experimental conditions were like those used to isolate active ARHGEF17 with the exception that lysis buffer contained 5 mM MgCl_2_.

### Western blotting, GST pull-down assays, and immunoprecipitation

Proteins were detected by Western blot as previously described (19, 82). ARHGEF17 constructs fused to GST were isolated by pull down with Glutathione Sepharose beads. For immunoprecipitation, total cell lysates containing either FLAG-ARHGEF17 or FLAG-Gβ1γ2 were mixed with 150 μg anti-FLAG antibodies and incubated overnight at 4 °C with constant shaking. Immunocomplexes were isolated with 25 μl protein-G agarose and revealed by Western blot (19, 82).

### Lentivirus generation and ARHGEF17 knockdown

Lentiviral pTRIPZ and pGIPZ constructs were obtained with the following targeting sequences: sh1-GEF17: TGCTGTTGACAGTGAGCGCGCCTGCCACCTTTACACCTATTAGTGAAGCCACAGATGTAATAGGTGTAAAGGTGGCAGGCATGCCTACTGCCTCGGA and sh2-GEF17:TGCTGTTGACAGTGAGCGGTACCACCCTGAAACGAAATAGTGAAGCCACAGATGTATTTCGTTTCAGGGTGGTACTGCCTACTGCCTCGGA cloned as XhoI/EcoRI and MluI/EcoRI, respectively. Knockdown efficiency was confirmed by transfection using NIH3T3 mouse fibroblasts. Selected clones were used to produce lentiviruses in HEK 293T cells. Briefly, cells were transfected with selected clones and the PAX2 and VSVG plasmids using Lipofectamine 2000. Fresh supernatants containing lentiviruses were used to infect LAP0297 cells, subjected to two cycles of infection within 6 h. Cells were selected with 3 μg/ml puromycin for 2 weeks. Knockdown efficiency was confirmed by Western blot.

### Cell migration and invasion

Cell migration experiments were done as previously described (50). In brief, confluent LAP0297 cells grown in 0.02% gelatin-pretreated 12-well plates were fasted for 24 h in serum-free DMEM. Two hours prior stimulation, cells were treated with 12 μM mitomycin. Migration started by scraping with a 10 μl tip and stimulated with 5 μM LPA, 50 ng/ml SDF-1, or 1 μM S1P; FBS was used as control. Cells were left migrating for 16 h, washed with PBS, fixed with formaldehyde, and stained with crystal violet. Cell migration was quantified with the ImageJ tool (nih.gov): MRI_wound_healing_tool.ijm. For invasion experiments, 1 × 10^5^ cells were seeded on top of 3 mg/ml Matrigel-coated transwell chambers (Corning Incorporated; catalog no.: 3422) and stimulated with 5 μM LPA for 24 h.

### Fluorescence microscopy and F-actin analysis

PAE cells seeded at subconfluence on 0.02% gelatin-pretreated coverslips were transfected with EGFP-tagged ARHGEF17 constructs in the presence or the absence of Gβγ, as indicated in the legends to the figures. F-actin was stained with phalloidin. Images were taken with a Nikon Ti-E inverted fluorescence microscope. Quantitative analysis of F-actin 8 bit pictures was done with ImageJ software. Threshold was adjusted from 90 to 255, and fluorescence intensity was measured on delimited areas of interest. At least 30 cells by condition were analyzed.

### Colocalization analysis of EGFP-ARHGEF17 with mCherry-Gβγ

PAE cells were transfected with EGFP-ARHGEF17 and mCherry-CAAX or EGFP-ARHGEF17 and mCherry-Gβγ. Cells were fasted for 16 h and fixed with paraformaldehyde to capture the images in a Leica confocal laser scanning microscope TCS SP8 using a 63× 1.4 oil immersion objective. Colocalization was analyzed with FIJI-ImageJ software using the Coloc2 plugin.

### Analysis of ARHGEF17 localization in LPA-stimulated PAE cells

PAE cells cotransfected with EGFP-ARHGEF17 and mCherry-CAAX were fasted for 16 h and subsequently stimulated with 5 μM LPA for 30 min. Cells were fixed with paraformaldehyde and visualized in a TCS SP8 Leica confocal laser scanning microscope using a 63× 1.4 oil immersion objective. Percentage of EGFP-ARHGEF17 at the cell periphery was calculated by determining the total intensity of fluorescence and the fluorescence at the cell perimeter, using the FIJI-ImageJ software. Cell perimeter was obtained by subtracting the central area of the cells delimited taking the mCherry-CAAX image as reference, which was converted to binary image, eroded and subtracted from the initial picture to obtain an image of the cell perimeter.

### Mouse tumor model

EGFP-LAP0297 cells (either sh-Control or sh-GEF17) were suspended at 106/100 μl in 4.5 mg/ml Matrigel (Corning Matrigel Growth Factor Reduced Basement Membrane Matrix, LDEV-free; catalog no.: 354230) and inoculated in the dorsal region of immunocompetent 6- to 8-week-old male FVB mice. Tumor size was measured with caliper, and volume was calculated with the equation: width × length^2^ × π/6 (50, 84).

Metastatic dissemination of LAP0297 (sh-Control or sh-GEF17) cells, 5 × 105/100 μl DMEM injected into the tail vein of FVB mice, was analyzed 15 days postinoculation. Mice were euthanized, and superficial metastases on the ventral and dorsal areas of the lungs were counted by two different experimenters. Pictures were taken in a SMZ25-Nikon stereo microscope. Noninoculated animals served as control. All procedures were approved by UPEAL-Cinvestav Ethical Committee (protocols 33–13 and 0205–16).

### NSCLC TCGA datasets

Percentage of cases with ARHGEF17 amplification (AMP) and wildtype, organized according to their tumor stage, were analyzed by Chi-squared test at the cBioPortal platform (https://www.cbioportal.org/). ARHGEF17 and ENPP2 expression/survival curves in NSCLC patients were analyzed at The Human Protein Atlas platform (https://www.proteinatlas.org/) selecting them according to their clinical stage. TCGA datasets contain 994 patients, including 500 lung adenocarcinoma patients and 494 lung squamous cell carcinoma patients. Only 12 cases did not have clinical information. ARHGEF17 and ENPP2 expression subgroups are indicated in Figures 1*C* and S1*B*, respectively. Maximally separated Kaplan–Meier plots are presented. *p* Values were calculated using Log-rank (Mantel Cox) test with GraphPad Prism 6 (GraphPad Software, Inc).

Expression of LPARs from RNA-Seq data in NSCLC TCGA datasets was analyzed at the cBioportal platform (https://www.cbioportal.org/). Number of tumor and normal samples are 994 and 110, respectively. *p* Values were calculated using unpaired *t* test with Welch's correction with GraphPad Prism 6. Coexpression of ENPP2 and ARHGEF17 in patients with stage II–IV tumors was analyzed at the cBioportal platform to assess their correlation by Spearman analysis.

### Membrane and cytosol fractionation of LAP0297 cells

LAP0297 cells were seeded in 10 cm dishes and serum-starved for 24 h, then were stimulated with 5 μM LPA for different times, as indicated in the corresponding figure legend before lysis. On ice, cells were scraped with the help of a gendarme and ice-cold PBS containing protease and phosphatase inhibitors. Cell lysates were obtained by three freeze–thaw cycles in liquid nitrogen and water at 35 °C. A fraction of total cell lysates were saved. Cytosolic and membrane fractions were obtained as previously described (82); Gβ1 and AKT1 served as membrane and cytosol markers, respectively. ARHGEF17 in the membrane fraction was normalized to the levels of Gβ1.

### Viability and proliferation experiments

For proliferation and viability experiments, 1500 and 50,000 cells were seeded in 96-well plates, respectively. Proliferation was assayed in cells grown overnight in DMEM containing 1% FBS followed by 48 h in DMEM with 10% serum. Viability was assessed in cells left overnight in DMEM with 1% FBS followed by 48 h in serum-free media. Cells grown in DMEM containing 1% FBS served as controls. At the end of the experiment, cells were incubated with 110 μl of 3-(4,5-dimethylthiazol-2-yl)-2,5-diphenyl-2H-tetrazolium bromide (50 μg/ml; Sigma–Aldrich) for 4 h at 37 °C. Subsequently, 100 μl of dimethyl sulfoxide was added, and absorbance was measured at 595 nm.

### Statistical analysis

All data are represented as mean ± SEM of at least three independent biological experiments. Graphic representation of data and statistical analysis by *t* test, Mann–Whitney *U* test, one or two-way ANOVA, followed Dunnett's or Tukey's test, were done with GraphPad Prism, version 6.05 software, as indicated in the legends to the figures. Significant difference was considered for values of *p* < 0.05.

## Data availability

All data are contained within the article.

## Supporting information

This article contains supporting information.

## Conflict of interest

The authors declare that they have no conflicts of interest with the contents of this article.
