# Supplementary material for: Gβγ mediates activation of Rho guanine nucleotide exchange factor ARHGEF17 that promotes metastatic lung cancer progression
Source: J Biol Chem. 2021 Nov 20;298(1):101440. doi: 10.1016/j.jbc.2021.101440 (PMC8703085; doi:10.1016/j.jbc.2021.101440)
Supplement: Figures S1 and S2 [file mmc1.pdf]

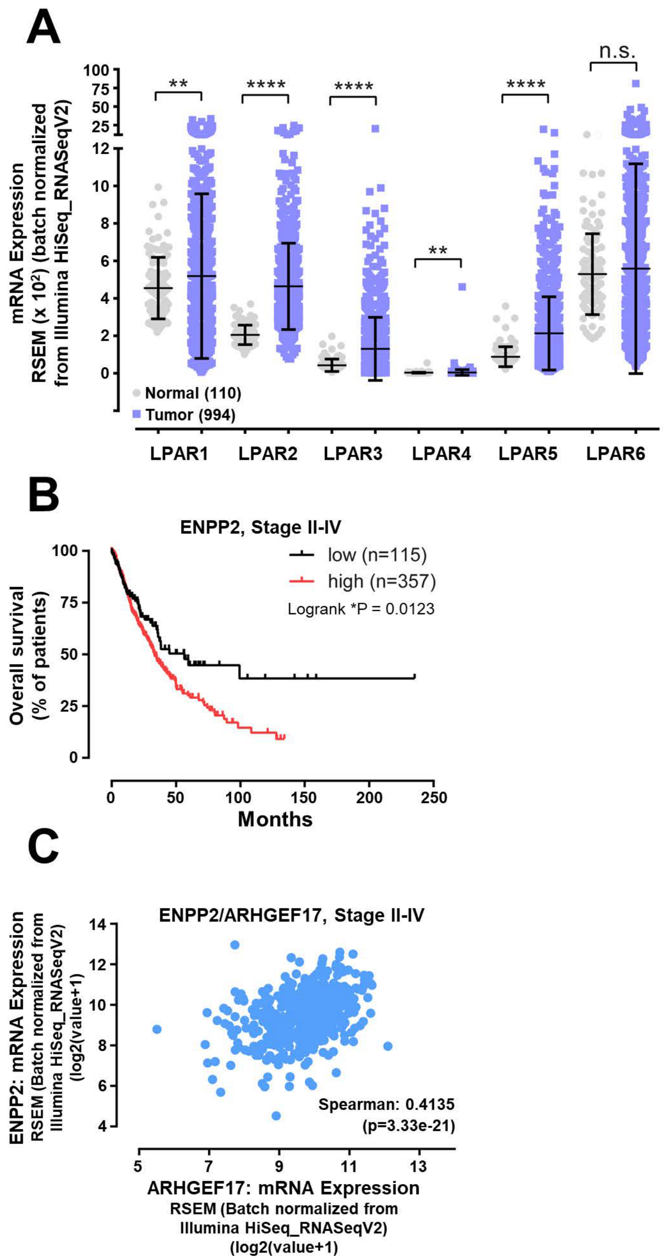

Supplementary Figure 1.
A) Expression of LPA receptors (RNAseq) in NSCLC TCGA datasets analyzed at the cBioPortal platform. Graph represents mean±SEM; ** $p<0.05$, **** $p<0.0001$.
B) Maximally separated Kaplan-Meier plots represent overall survival of stage II-IV NSCLC patients with high and low ENPP2 expression.
C) ENPP2:ARHGEF17 expression in stage II-IV NSCLC TCGA patients. Coexpression was analyzed at the cBioPortal platform.

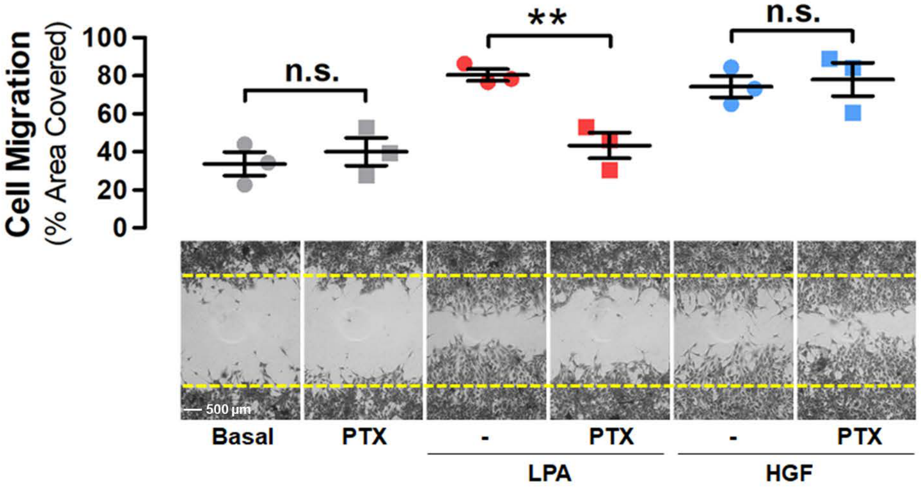

Supplementary Figure 2.
LPA-induced LAP0297 cell migration was inhibited with persussis toxin (PTX), but HGF-induced migration was not. Before wound closure assays, cells were preincubated overnight with 100ng/mL PTX. Cell migration was stimulated with 5µM LPA or 10ng/mL HGF. Data represent 3 independent experiments, t-test.
